# The HLH-6 Transcription Factor Regulates *C. elegans* Pharyngeal Gland Development and Function

**DOI:** 10.1371/journal.pgen.1000222

**Published:** 2008-10-17

**Authors:** Ryan B. Smit, Ralf Schnabel, Jeb Gaudet

**Affiliations:** 1 Genes and Development Research Group, Department of Biochemistry and Molecular Biology, University of Calgary, Calgary, Alberta, Canada; 2 Institut für Genetik, Technische Universität Braunschweig, Braunschweig, Germany; 3 Department of Medical Genetics, University of Calgary, Calgary, Alberta, Canada; Huntsman Cancer Institute, United States of America

## Abstract

The *Caenorhabditis elegans* pharynx (or foregut) functions as a pump that draws in food (bacteria) from the environment. While the “organ identity factor” PHA-4 is critical for formation of the *C. elegans* pharynx as a whole, little is known about the specification of distinct cell types within the pharynx. Here, we use a combination of bioinformatics, molecular biology, and genetics to identify a helix-loop-helix transcription factor (HLH-6) as a critical regulator of pharyngeal gland development. HLH-6 is required for expression of a number of gland-specific genes, acting through a discrete *cis*-regulatory element named PGM1 (Pharyngeal Gland Motif 1). *hlh-6* mutants exhibit a frequent loss of a subset of glands, while the remaining glands have impaired activity, indicating a role for *hlh-6* in both gland development and function. Interestingly, *hlh-6* mutants are also feeding defective, ascribing a biological function for the glands. Pharyngeal pumping in *hlh-6* mutants is normal, but *hlh-6* mutants lack expression of a class of mucin-related proteins that are normally secreted by pharyngeal glands and line the pharyngeal cuticle. An interesting possibility is that one function of pharyngeal glands is to secrete a pharyngeal lining that ensures efficient transport of food along the pharyngeal lumen.

## Author Summary

To make an organ, cells must be instructed to be part of a common structure yet must also be assigned specific roles or identities within that structure. For example, the stomach contains a variety of different kinds of cells, including muscles, nerves, and glands. This same complexity is seen even in relatively simple organs, like the pharynx (foregut) of the nematode *C. elegans*. The pharynx is a neuromuscular organ that pumps in food (bacteria) from the environment. This organ is relatively simple (containing only 80 cells) yet contains five distinct kinds of cells. How these different cells are specified is unclear but likely involves combinations of developmental regulators known as transcription factors. Here, we examine one cell type, the pharyngeal glands, and identify a key regulator of their development, the transcription factor HLH-6. Interestingly, HLH-6 is closely related to a mammalian transcription factor, Sgn1, which is involved in development of mammalian salivary glands, suggesting that *C. elegans* pharyngeal glands are evolutionarily related to mammalian salivary glands. A further connection is that the pharyngeal glands of *C. elegans* appear to be required for efficient feeding, possibly by secreting mucin-like proteins that ensure the smooth passage of food along the digestive tract.

## Introduction

**Figure 1:**
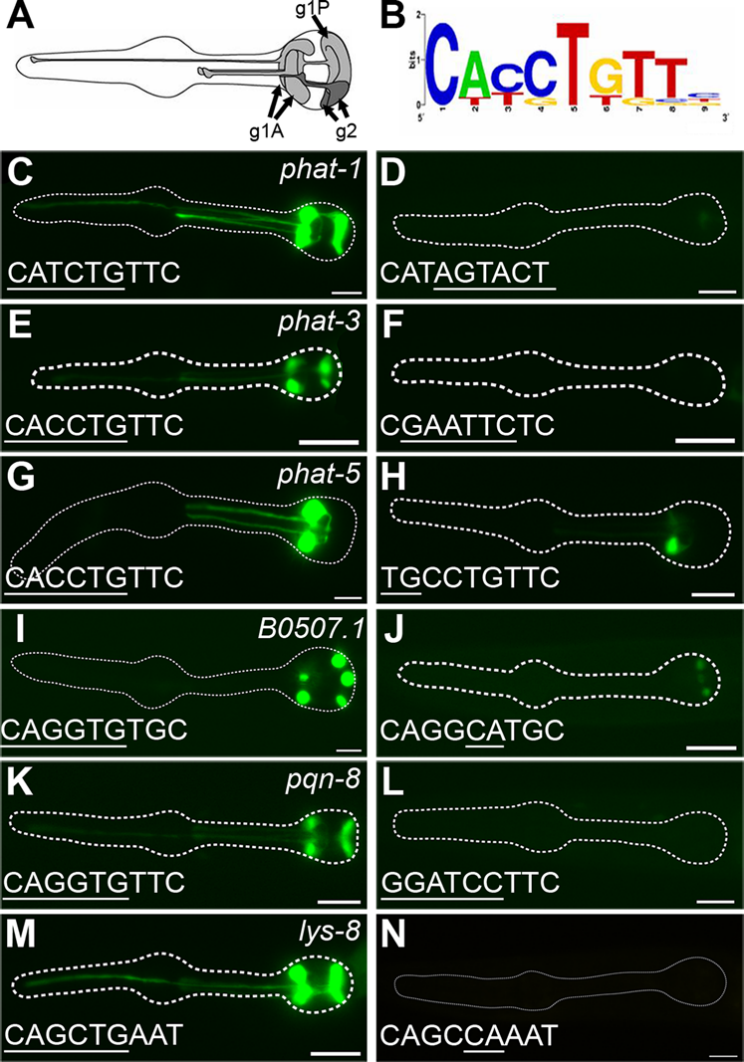
PGM1 is required for expression of some pharyngeal gland genes. (A) Diagram of pharynx, highlighting the pharyngeal glands, modified from [9]. (B) WebLogo [80] of computationally identified PGM1. (C–N) Fluorescence micrographs of gland-expressed GFP or YFP reporters with wild-type promoter sequence (left column) or promoter sequence in which PGM1 is mutated (right column). In wild-type sequences (left) the E-box is underlined and in mutant sequences (right) the mutation is underlined. Anterior is at left and the pharynx is outlined. Scale bars represent 10 µm.

An important question in the study of organ development is how different cells are instructed to become part of a common structure and yet are also specified to have a distinct identity within that structure. This problem is well-illustrated in the pharynx of the nematode *C. elegans*. The pharynx is a small (80 cells) neuromuscular organ that pumps food (bacteria) in from the environment and initiates digestion (Figure 1A). It contains five different cell types (muscles, epithelia, neurons, marginal cells and glands) that are not restricted by their lineal origins. Recruitment of cells to the pharynx involves the “organ identity factor” PHA-4 (the *C. elegans* FoxA ortholog), which is required for cells to adopt a pharyngeal identity [1]–[3]. Available data supports a model in which PHA-4 directly regulates most or all genes that are expressed in the pharynx [4]. However, PHA-4 alone cannot be responsible for all aspects of organ development and must function with other factors to control the various sub-programs of pharyngeal organogenesis, such as specification of the distinct cell types. Aside from the involvement of PHA-4, little is known about the specification and development of any of the distinct pharyngeal cell types, though regulators of pharyngeal muscle development have been identified [5]–[8].

In this work, we chose to examine development of the pharyngeal glands, one of five cell types in the pharynx [9]. We chose this cell type for three reasons: first, nothing is known about regulation of gland gene expression nor about the specification of the glands (aside from the general involvement of PHA-4). Second, the function of the glands in *C. elegans* is poorly understood, although proposed roles include initiation of digestion, molting of the pharyngeal cuticle and resistance to pathogenic bacteria [9]–[12] and the digestive tract glands of parasitic nematodes are known to play crucial roles in host-parasite interactions (reviewed in [13]). Third, several genes with gland-specific expression have been identified, based on a combination of microarray and *in situ* hybridization data [14]–[16].

The pharyngeal glands are five cells in the posterior bulb of the pharynx with cellular projections that open into the pharyngeal lumen at discrete points along the length of the pharynx [9] (Figure 1A). The glands are further divided into two sub-groups, g1 and g2, based on their appearance in electron micrographs, though the significance of these sub-types is not known.

Given recent advances in computational biology and genomics, one powerful approach to exploring the issue of cell type specification is to identify a group of co-expressed (and presumably co-regulated) genes and from this group identify shared regulatory elements. These elements can then be used as tools for determining and characterizing the relevant *trans*-acting factor(s).

Here we identify both a *cis*-acting regulatory element (PGM1) and the corresponding *trans*-acting factor (HLH-6) that are together necessary and sufficient for pharyngeal gland-specific gene expression. We further show that elimination of HLH-6 results in the loss of a subset of pharyngeal glands, disrupted function of the remaining glands and defects in feeding that lead to partial starvation. Based on our analysis of *hlh-6* mutants, we propose that one function of pharyngeal glands is to assist in the transport of food through the pharyngeal lumen. The glands secrete mucin-like proteins that line the pharyngeal lumen, which possibly lubricate the tract to ensure efficient passage of bacteria. These results not only demonstrate an important function of the pharyngeal glands, but also illustrate evolutionary conservation of foregut gland function, as both *C. elegans* pharyngeal glands and a component of the vertebrate foregut, the salivary glands, have roles in ensuring efficient transport of food through the front end of the digestive tract [17].

## Results

### Identification of a Candidate *cis*-Acting Element in Pharyngeal Gland-Expressed Genes

To investigate regulation of pharyngeal gland development, we first searched for *cis*-regulatory elements in the promoters of gland-expressed genes. Co-expressed genes often share common *cis*-acting regulatory elements, and identification of elements required for gland expression could lead to the identification of the corresponding *trans*-acting factors. We began with a list of fourteen confirmed and probable gland-specific genes, based on previous work [14] (Table 1). Twelve of these fourteen genes are predicted to encode proteins whose only recognizable features are a signal peptide and multiple copies of the ShK motif, a cysteine-rich sequence first described in metridin toxin from the sea anemone [18]. Proteins containing only ShK motifs appear to be gland-specific, while proteins containing ShK motifs in the presence of other recognizable domains (such as astacin in NAS-14 or tyrosinase in TYR-1) are not gland-specific [14]. We modified the original list of fourteen genes by excluding one gene (*C14C6.5*) that contains motifs in addition to ShK and also lacks supporting expression data. We also added one gene (*T10B10.6*) that encodes an ShK protein and is expressed solely in pharyngeal glands according to available *in situ* hybridization data [15],[16] (Table 1). We will refer to ShK-encoding genes with confirmed gland-specific expression as *phat* genes, for pharyngeal gland toxin-related.

**Table 1:** The list of gland-expressed genes.

Gene	Name	Expression	Supporting Evidence	PGM1/HLH-6 Dependent?	Prominent Motifs
**B0507.1**		All pharyngeal glands	Reporter, *in situ* hybridization	YES	EGF-like (×2), Worm-specific repeat type 1 (×2)
**C46H11.8**	*phat-1*	All pharyngeal glands	Reporter*, *in situ* hybridization	YES	ShK (×4)
C46H11.9	*phat-2*	All pharyngeal glands	*in situ* hybridization	ND	ShK (×3)
**C49G7.4**	*phat-3*	All pharyngeal glands	Reporter*, *in situ* hybridization	YES	ShK (×3)
T05B4.3	*phat-4*	All pharyngeal glands	*in situ* hybridization	ND	ShK (×3)
**T05B4.11**	*phat-5*	Anterior-most pharyngeal glands (g1A)	Reporter, *in situ* hybridization	YES	ShK (×3)
T10B10.6	*phat-6*	Anterior-most pharyngeal glands	*in situ* hybridization	ND	ShK (×1)
T20G5.7	*dod-6*	All pharyngeal glands	*in situ* hybridization	ND	ShK (×1)
F07C4.11		Probable gland	Microarray	ND	ShK (×2)
F41G3.10		Probable gland	Microarray	ND	ShK (×3)
M153.3		Probable gland	Microarray	ND	ShK (×2)
T05B4.8		Probable gland	Microarray	ND	ShK (×3)
T05B4.12		Probable gland	Microarray	ND	ShK (×3)
T05B4.13		Probable gland	Microarray	ND	ShK (×3)

Supporting *in situ* hybridization data is from NEXTDB [15],[16]. Microarray data [27] indicates probable pharyngeal expression, though not necessarily gland-specific. * = reporter expression previously described [14]. Dependence on both PGM1 and HLH-6 is experimentally verified for four genes on this list, indicated in bold.

To verify the quality of the list of fourteen genes, we constructed GFP or YFP reporters for four of the genes (two of which were previously reported; [14]) and found that all four were expressed specifically in pharyngeal glands (Figure 1 and Table 1). Of the four genes, three (*B0507.1*, *phat-1*, and *phat-3*) were expressed in all five glands (Figure 1C,E,I), while *phat-5* was only expressed in the two anterior-most glands, the left and right g1A cells (g1AR and g1AL; Figure 1G). Previous reports have suggested that the g1AR and g1P cells are fused [9], yet we see no passage of *phat-5*-expressed YFP from g1AR to g1P, suggesting either that YFP is restricted from diffusing between these cells or that the two cells are not fused.

By searching the upstream 500 bp (relative to the ATG) of the fourteen gland genes using the Improbizer program [14] for shared sequence motifs, we identified one candidate gland-specific *cis*-acting element, which we named PGM1 (for Pharyngeal Gland Motif 1; Figure 1B). This size of promoter was justified because many of the gland genes have neighboring genes within 500 bp upstream, consistent with the observation that *C. elegans* promoters are generally small [19],[20]. PGM1 was the only motif identified by Improbizer that had a position weight matrix score higher than any of the motifs generated in control runs (See Materials and Methods), suggesting that it might be a functional regulatory element. In addition, PGM1 appeared to be enriched in the promoters of gland-expressed genes, as these promoters were four times more likely to contain significant occurrences of PGM1 (12/14 = 86%) than a control set of promoters from pharyngeal (but not gland-specific) genes (20/96 = 21%) (Table S1).

### PGM1 Is Necessary for Expression in Pharyngeal Glands

Analysis of PGM1 in the context of pharyngeal gland-specific promoters demonstrated that PGM1 was required for expression. Site-directed mutations in PGM1 sequences eliminated expression of *phat-1* and *phat-3* reporters, and greatly reduced expression of *B0507.1* and *phat-5* reporters (Figure 1C–J). The promoter of *phat-5* has one other potential occurrence of PGM1 that could account for its residual activity (at −118 bp; Figure S1). The *B0507.1* promoter has no other apparent PGM1 sequences, suggesting that the remainder of its expression is dependent on an as yet unidentified *cis*-regulatory motif. Together, these results suggested that PGM1 is necessary for the high level expression of a subset of genes in pharyngeal glands. We queried other gland-expressed genes to determine whether they also required PGM1 for expression. We analyzed the expression of two genes that were not part of our original data set, but that were reported to be expressed in glands: *pqn-8* and *lys-8* [21],[22]. The *pqn-8* reporter was expressed exclusively in pharyngeal glands whereas the *lys-8* reporter was expressed in pharyngeal glands and the intestine, as reported (Figure 1K, M). Mutation of a PGM1 sequence in the *pqn-8* promoter completely abolished expression (Figure 1 K–L). The *lys-8* promoter had three potential PGM1 sites at −180, −452 and −581 bp relative to the ATG (Figure S1). Two of these sequences (at −180 and −452) are not required for expression in pharyngeal glands (data not shown), while mutation of the third site (−581 bp) resulted in a loss of expression (Figure 1M–N).

Not all pharyngeal gland genes contain identifiable PGM1 sequences. In a search for additional pharyngeal gland genes based on *in situ* hybridization data [15],[16], we identified *Y8A9A.2* as a probable gland-expressed gene that does not contain a PGM1 sequence in its promoter. Expression in pharyngeal glands was verified with a transcriptional *Y8A9A.2*::*GFP* reporter containing 2000 bp of upstream sequence (relative to the ATG) (Figure S2). This reporter does not contain any sequence that resembles a PGM1 site, suggesting that its expression is PGM1 independent or that there is an occurrence of PGM1 that is too divergent to be recognizable. Based on further analysis (below), *Y8A9A.2*::*GFP* expression is likely to be PGM1-independent.

**Figure 2:**
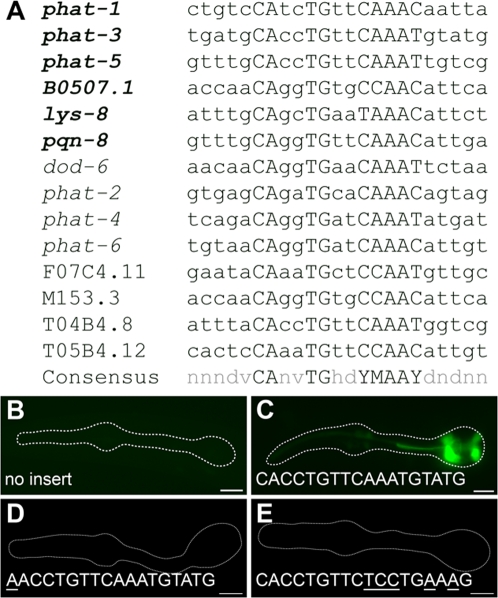
The extended PGM1 is sufficient for gland-specific expression. (A) Alignment of PGM1 occurrences in the promoters of gland-expressed genes. Expression of genes in bold is experimentally verified to be both PGM1 and HLH-6 dependent. (B–E) Fluorescence micrographs of GFP enhancer constructs containing (B) no insert, (C) three tandem copies of the extended PGM1, (D) three tandem copies of the extended PGM1 in which the E-box has been mutated and (E) three tandem copies of the extended PGM1 in which sequence flanking the E-box has been altered. Anterior is at left and the pharynx is outlined. Scale bars represent 10 µm.

Closer examination of PGM1 revealed that it contains an E-box (CAnnTG), the consensus binding site for basic helix-loop-helix (bHLH) transcription factors [23]. Mutations that specifically disrupt the E-box sequence eliminate PGM1 activity (Figure 1). However, the E-box is not sufficient for PGM1 activity: mutation of sequence flanking the E-box in the *phat-1* reporter resulted in a significant loss of expression (data not shown), suggesting that an extended sequence is required for activity. Alignment of the functionally defined PGM1 sequences revealed an extended consensus of CAnvTGhdYMAAY (where V = A, C or G, H = A, C or T, D = A, G or T, M = A or C, and Y = C or T; Figure 2A). This extended consensus is present in all 12 of the 14 genes in our initial list that contained PGM1 (Figure 2A). The functionally defined consensus may represent either an extended binding preference for the relevant *trans*-acting factor or the juxtaposition of binding sites for two (or more) distinct factors.

### The Extended PGM1 Is Necessary and Sufficient for Expression in Pharyngeal Glands

Given that PGM1 is necessary for expression of many genes in pharyngeal glands, we next asked whether PGM1 was also sufficient for gland expression. Indeed, three tandem copies of the PGM1 sequence from *phat-3* placed upstream of a “promoter-less” reporter (to make the “3×PGM1” construct) was sufficient to activate pharyngeal gland expression in 78% (31/40) of transgenic animals (Figure 2B–C). A fraction of these animals (7/31) also showed weak expression in the I3 pharyngeal neuron, a sister cell of the g1P gland [24]. These results indicate that PGM1 is a pharyngeal gland-specific enhancer element, and further suggests that PGM1 is a binding site for one or more transcription factors that function in pharyngeal glands.

Given the apparent extended consensus sequence for PGM1, we performed additional enhancer tests to determine what portions of PGM1 were required for its activity. We first tested a version of the 3×PGM1 plasmid in which all three copies of the E-box were changed from CAnnTG to AAnnTG. This construct (3×PGM1ΔE) showed no expression in transgenics, indicating (as above) that the E-box was required for PGM1 activity (Figure 2D). We next tested an enhancer in which sequence flanking the E-box was altered (3×PGM1Δflank) and found that this sequence was also required for PGM1 activity (Figure 2E), demonstrating that the E-box is necessary but not sufficient for PGM1 activity.

### HLH-6 Functions through PGM1

Since PGM1 activity is dependent on an E-box sequence, our search for the relevant *trans*-acting factor(s) began with bHLH proteins. bHLH proteins typically bind to DNA as heterodimers, composed of a ubiquitous “Class I” subunit and a tissue-restricted “Class II” partner (reviewed in [25]). In *C. elegans*, the sole Class I bHLH is encoded by *hlh-2* [26], which is expressed in many cells throughout development, including the glands. To identify the relevant Class II bHLH, we examined data from microarray experiments that identified candidate pharynx-expressed genes [4],[27], including three Class II bHLHs: *hlh-3*, *hlh-6* and *hlh-8*. Both *hlh-3* and *hlh-8* are expressed exclusively in non-pharyngeal tissue (in neurons and muscles, respectively; [28]) suggesting that they are false positives with respect to the microarray data and are thus unlikely to function through PGM1. At the time of our analysis, *hlh-6* was uncharacterized and was therefore a candidate PGM1 *trans*-acting factor.

**Figure 3:**
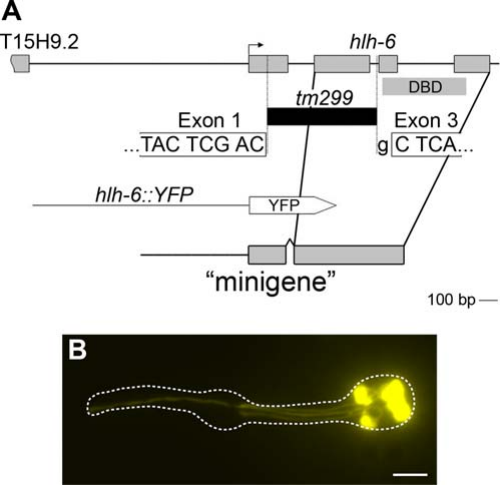
*hlh-6* is expressed in pharyngeal glands. (A) Schematic of the genomic region containing *hlh-6*. The position of the deletion allele *tm299* is indicated. The portion of *hlh-6* encoding the DNA Binding Domain (DBD) is shown as is the *hlh-6* “minigene”, which rescues all aspects of the *hlh-6* mutant phenotype. (B) Expression of the *hlh-6::YFP* reporter, containing 1175 bp (of 1190 bp) of intergenic sequence from the ATG of *hlh-6* to just downstream of the stop codon of the next upstream gene, *T15H9.2*. Anterior is at left and the pharynx is outlined. Scale bar represents 10 µm.

To examine the involvement of *hlh-6* in PGM1 activity, we first determined the expression of a transcriptional reporter that included almost all intergenic sequence (1175 bp of 1190 bp) between *hlh-6* and its nearest upstream neighbour, *T15H9.2*. We found that *hlh-6::YFP* was expressed strongly and specifically in the pharyngeal glands (98% of transgenics), with occasional (12%), weak expression in the pharyngeal neuron I3 (Figure 3). Expression was first detectable shortly after the terminal cell division that gives rise to pharyngeal glands (bean stage embryos) and persisted throughout the life cycle in all five pharyngeal glands. Because PGM1 and *hlh-6* both appear to be active in pharyngeal glands and because PGM1 contains a bHLH binding site, we hypothesized that HLH-6 is the cognate *trans*-acting factor for PGM1.

**Figure 4:**
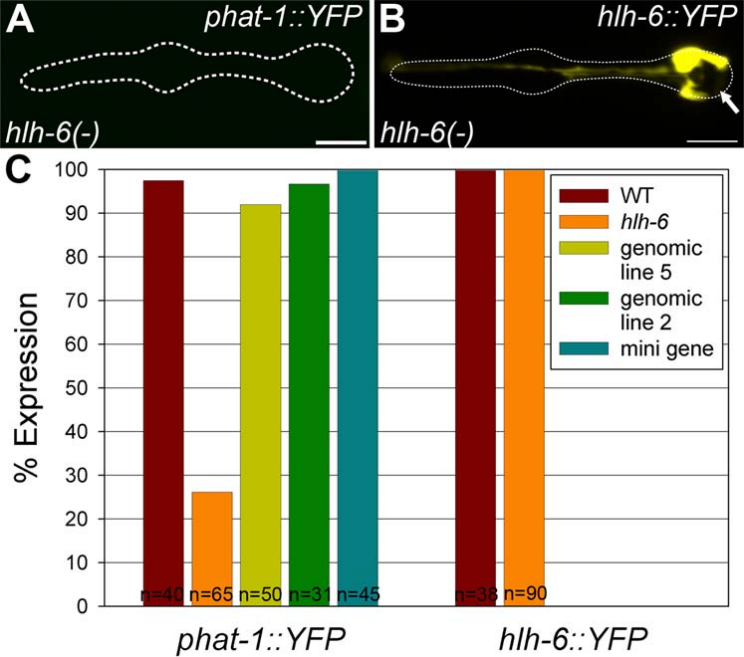
*hlh-6* is required for PGM1 activity. (A) An example of *hlh-6* mutants where expression of *phat-1::YFP* is not visible. (B) An example of *hlh-6* mutants where expression of *hlh-6::YFP* is visible only in g1 cells. The absence of the g2 cells is indicated by the arrow. Anterior is at left and the pharynx is outlined. Scale bars represent 10 µm. (C) Quantitation of the number of animals expressing each reporter in *hlh-6* mutants. For the *phat-1::YFP* reporter in wildtype and *hlh-6* mutants, only one transgenic line was scored but the same array was used in both genotypes. Two lines of the genomic rescue were scored for *phat-1::YFP* expression (lines 5 and 2). Only one line of minigene rescue was scored. Number of animals scored is indicated.

We determined that HLH-6 is required for PGM1 activity by demonstrating that PGM1-dependent reporters were not expressed in *hlh-6(tm299)* mutants. The deletion mutant *hlh-6(tm299)* (generously provided by S. Mitani; [29]) is a probable null, as it removes 595 bp from *hlh-6*, including all but one nucleotide from the second intron, resulting in a frameshift (Figure 3A). The mutation is homozygous viable (see Materials and Methods), which allowed us to examine gland reporter expression in these mutants. We found that expression of 6/6 gland reporters (*phat-1*, *phat-3*, *phat-5*, *B0507.1*, *pqn-8* and *lys-8*) was significantly reduced in *hlh-6* animals (Figure 4A,C; Figure S2). For example, only 26% of *hlh-6* mutants had visible *phat-1::YFP* expression (n = 65), and this expression was significantly weaker than the expression seen in 100% of wild type animals. Four of the other gland reporters showed a similar loss of expression in *hlh-6* mutants. Expression of the *B0507.1* reporter was less affected than the others, consistent with it being only partially PGM1 dependent. Likewise, expression of *Y8A9A.2::GFP*, which lacks an identifiable PGM1 sequence, was unaffected in *hlh-6* mutants (Figure S2). There is thus a perfect correlation between PGM1-dependent gene expression and *hlh-6*-dependent gene expression, implying that HLH-6 is acting directly on the reporters rather than earlier in the pathway of gland specification.

To confirm that loss of reporter expression was due to the *hlh-6* mutation, we performed transgenic rescue with either genomic *hlh-6* or an *hlh-6* “minigene”. The genomic fragment contains *hlh-6* and 2030 bp upstream of the ATG (including 840 bp of the upstream neighbour, *T15H9.2*) and 60 bp downstream of the predicted stop codon. The minigene construct consists of 568 bp of promoter sequence fused to *hlh-6* cDNA containing a synthetic intron (Figure 3). The 568 bp promoter fragment is only active in pharyngeal glands [30], so the *hlh-6* minigene is expressed only in pharyngeal glands. Both genomic and minigene versions of *hlh-6* rescued *phat-1* reporter expression in *hlh-6* mutants (Figure 4C).

Together, the above three lines of evidence indicate that the bHLH transcription factor encoded by *hlh-6* functions through PGM1. First, PGM1 activity depends on an E-box, the canonical binding site for bHLH transcription factors. Second, the expression patterns of *hlh-6* and the PGM1 enhancer are identical. Third, *hlh-6* is required for PGM1-dependent reporter activity.

### *hlh-6* Mutants Frequently Lack g2 Glands

Given that *hlh-6* was required for expression of PGM1-dependent genes, we next examined *hlh-6* mutants to determine the effect on pharyngeal gland development using our *hlh-6* reporter. Expression of *hlh-6* is not critically dependent on *hlh-6*, though *hlh-6* shows weak autoactivation [30]. An integrated *hlh-6::YFP* reporter is expressed in 100% of *hlh-6* mutants (Figure 4C). However, in 84% of *hlh-6* mutants (n = 90), expression was observed in only three gland cells, rather than the expected five (Figure 4B). This finding was verified with a nuclear-localized fluorescent reporter (data not shown). Based on the position and morphology of expressing cells, it appeared that the three g1 glands (g1AR, g1AL and g1P) were present, while the two g2 cells were either missing or failed to express all gland reporters (*hlh-6::YFP*, *phat-1::YFP*, *B0507.1::GFP*, *et al.*).

The apparent absence of g2 glands in *hlh-6* mutants could be explained by three possibilities: first, the g2 glands may undergo apoptosis; second, the cells may be mis-specified and adopt an alternate fate; third, the cells may persist as undifferentiated cells. The sister cells of the g2 glands undergo apoptosis in normal development [24] and so we tested whether blocking apoptosis with a mutation in *ced-3* would restore g2 glands. Strong loss-of-function mutations in *ced-3* result in the survival of all cells that normally undergo programmed cell death [31],[32]. However, only 9% of *hlh-6*; *ced-3* double mutants (n = 32) expressed the *hlh-6::YFP* reporter in g2 cells, comparable to the expression in *hlh-6* mutants, indicating that g2 glands are not restored by preventing apoptosis.

To address the possibility that g2 glands adopt an alternate cell fate, we performed nuclear counts in the back half of the posterior pharyngeal bulb where the g2 cells are normally located using a *pha-4* reporter, which is expressed in all pharyngeal nuclei except for some pharyngeal neurons [3]. There are 11 pharyngeal cells in this region (four muscles, three marginal cells, three glands and one neuron), 10–11 of which express *pha-4* post-embryonically (expression in the pharyngeal neuron in the posterior bulb is variable). We expected that *hlh-6* mutants would either have a wild type number of PHA-4-expressing cells or an average loss of ∼1.6 such cells (because ∼80% of *hlh-6* mutants do not have visible g2 cells). There was a significant decrease in *pha-4::GFP::HIS2B* expressing cells between wild type and *hlh-6* mutants (9.1 vs. 7.8, respectively, p<0.05), suggesting that either the presumptive g2 cells do not express *pha-4::GFP::HIS2B* or the cells are not present. Consistent with these cells not having a pharyngeal identity, we did not observe an increase in the numbers of other pharyngeal cell types, demonstrating that the presumptive g2 cells have not adopted an alternate pharyngeal identity (Figure S3). In the course of these nuclear counts, we also observed that the numbers of other types of pharyngeal nuclei were not affected in *hlh-6* mutants. In particular, pm6 cells, which are lineally-related to the g2 glands, were present and expressed the correct markers (data not shown). This suggests that the *hlh-6* mutation specifically affects glands and does not act in the differentiation of other pharyngeal cell types, as expected given the expression pattern of *hlh-6*.

**Figure 5:**
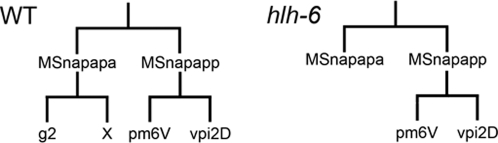
The g2 glands are not generated in *hlh-6* mutants. The lineages of the g2 glands in wild-type and *hlh-6* mutants. MSn is used because both the MSa and MSp cell give rise to a g2 cell. If n = a, the g2L cell is made (as well as pm6VL and vpi2DL) and if n = p, the g2R cell is made (and pm6VR and vpi2DR). The sister cell of g2 cell undergoes apoptosis (X) in wild-type animals.

The failure of the presumptive g2 cells to express any tested pharyngeal reporters implies that these cells were not present in *hlh-6* mutants. To explore this possibility, we followed the lineages that give rise to g2 in *hlh-6* mutant animals. In eight cases (73%), the immediate precursor to the g2 cell (MSnapapa) failed to undergo its terminal division, but remained in its usual position within the embryo (Figure 5). In one case, the grandmother of g2 failed to divide. Such a lineage defect would prevent formation of one of the pm6 muscles, though we do not see a loss of pm6 cells in *hlh-6* mutants. In the remaining two cases (18%), the g2 precursor underwent its normal division. Thus, in 82% of cases, the g2 cell failed to be generated, consistent with our observation that 84% of *hlh-6* mutants do not express *hlh-6* in g2 cells. Interestingly, PHA-4 expression is lost in the arrested g2 precursors, based on our counts of *pha-4::GFP::HIS2B* nuclei, yet PHA-4 must be normally expressed earlier in this lineage (i.e., in the g2 grandmother MSnapap), as no other pharyngeal cells (e.g., pm6 cells, which are cousins of the g2s) were missing. Formally, this result indicates that *hlh-6* is required for maintenance of *pha-4* expression in g2 cells, though the nature of this regulation is unclear.

### *hlh-6* Mutants Are Feeding Defective

**Figure 6:**
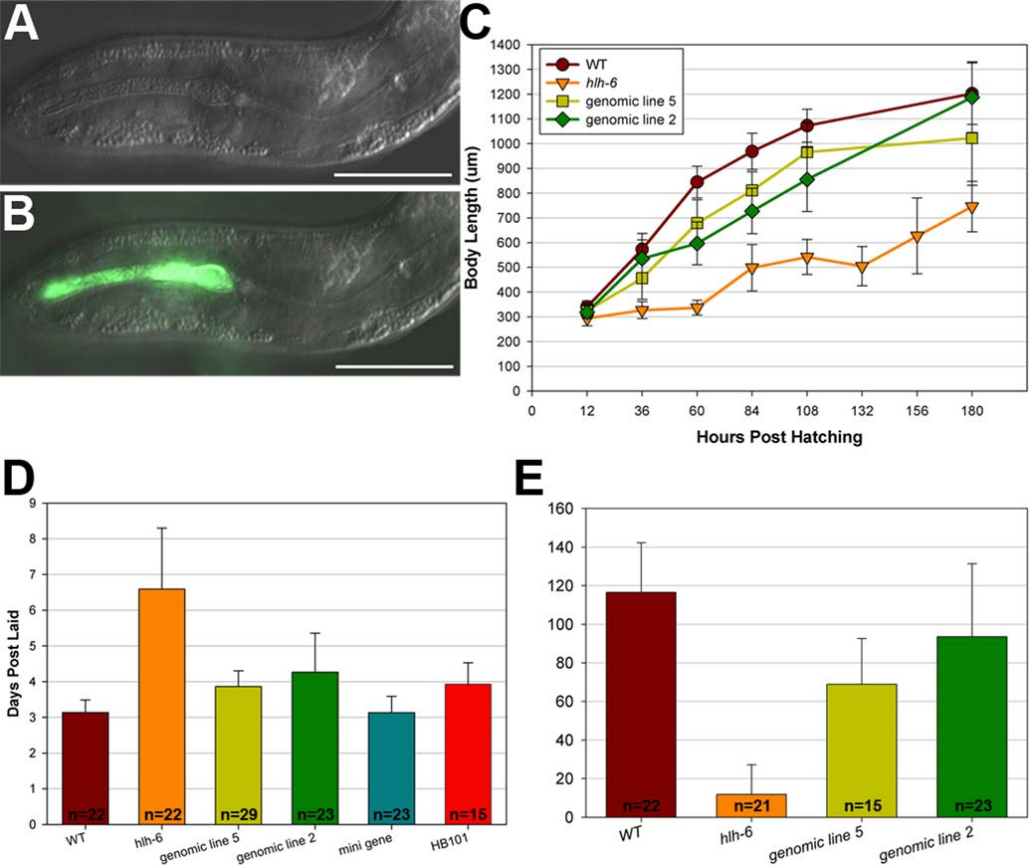
Phenotypic analysis of *hlh-6* mutants. (A–B) The stuffed pharynx phenotype of *hlh-6* mutants grown on OP50-GFP bacteria. (A) NDIC image, (B) merged NDIC and fluorescence image. Anterior is at left and scale bars represent 10 µm. (C–E) Assays for growth defects in wild-type, *hlh-6* mutants and *hlh-6* mutants rescued by either the *hlh-6* genomic fragment, the *hlh-6* minigene or by using the HB101 strain of *E. coli*. (C) Graph of body length versus time, (D) time to reach adulthood and (E) brood sizes. For the *hlh-6* mutants the L1 arrested animals are omitted. Error bars represent one standard deviation.

In addition to a loss of gland gene expression and defects in gland development, *hlh-6* animals display a variety of characteristics that indicate a starvation phenotype: partially penetrant larval arrest, slow growth, smaller body size and decreased brood size among those surviving to adulthood. On average, 32% (n = 105) of *hlh-6* mutants arrest as L1 larvae. The anterior pharyngeal lumen of arrested larvae is stuffed with bacteria (Figure 6A–B), indicating a failure of these animals to properly transport food along the pharyngeal lumen. Animals that develop beyond the L1 stage also exhibit signs indicative of starvation. First, *hlh-6* mutants are consistently smaller than wild-type worms of the same chronological age (Figure 6C). Adult *hlh-6* mutants are roughly half the length of wild-type adults (635±210 µm vs. 1202±124 µm, n = 23 and 14 respectively). *hlh-6* mutants also grow more slowly than control strains, taking more than twice as long to reach sexual maturity compared to controls (6.6±1.7 days vs. 3.1±0.4 days after embryos were collected, n = 22 and 22; Figure 6D). As adults, *hlh-6* mutants have dramatically smaller broods, laying an average of 11.9±15.4 eggs throughout their lifetime (n = 21) compared to the congenic control *rol-6 unc-4* strain (116.5±25.7 eggs, n = 22; Figure 6E). All aspects of the *hlh-6* mutant phenotype were rescued by either the *hlh-6* genomic fragment or the “minigene” constructs described previously (data not shown and Figure 6C–E), indicating that the phenotypes result from a loss of *hlh-6* activity in the pharyngeal glands. The larval arrest, small size, slow growth and low brood size are all characteristic of starvation and are observed in other mutants that are feeding defective, such as the *eat* mutants and animals with abnormal pharynx morphology [33],[34].

**Figure 7:**
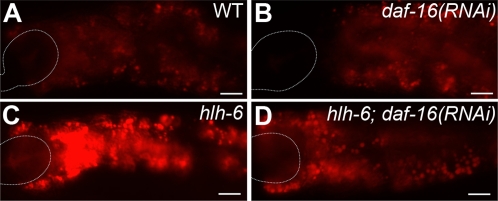
Staining of intestinal fat stores of *hlh-6* mutants. (A–D) Fluorescence images of animals grown in the presence of Nile Red. (A) wild type, (B) *daf-16(RNAi)*, (C) *hlh-6* and (D) *hlh-6*; *daf-16(RNAi)*. Anterior is at left and the pharynx is outlined. Scale bars represent 10 µm.

To further verify that *hlh-6* mutants are starved, we stained animals with the lipophilic dye Nile Red, which detects intestinal fat stores [35]. We consistently observed increased fat stores in *hlh-6* mutants compared to control strains (Figure 7A,C). Increased fat stores are observed with other feeding defective strains (e.g., *tph-1*; [35],[36]), reflecting a metabolic response to decreased nutrient availability or uptake. Other starvation mutants, however, such as *pha-2* and *pha-3*, have more severe feeding defects and exhibit decreased fat stores, possibly because food uptake is too low to provide nutrients to store as fat [34]. These results suggest that *hlh-6* mutants may not be as severely starved as *pha-2* and *pha-3* mutants. However, because Nile Red staining does not always correlate with fat levels, further investigation is required to verify this interpretation [37]. Increased fat storage in response to starvation requires the activity of the transcription factor DAF-16/FoxO (reviewed in [38]). Accordingly, *daf-16(RNAi)* suppressed the increased fat storage of *hlh-6* mutants, indicating that the starvation response of *hlh-6* animals acts through the canonical DAF-16-dependent pathway (Figure 7B,D). Control feeding with *GFP(RNAi)* did not affect Nile Red staining.

The starvation of *hlh-6* mutants can be rescued by providing an alternate food source. *C. elegans* are usually grown by feeding with the *E. coli* strain OP50, though feeding with the strain HB101 can rescue the starvation phenotype of some *eat* mutants, which appears to be easier for *C. elegans* to eat [39]. We found that the mutants grown on HB101 were not starved, exhibiting wild type growth rates and a suppression of larval arrest (Figure 6D and data not shown). Two factors that affect the ability of different food sources to rescue *eat* mutants are bacterial cell size and the relative “stickiness” of the cells [39]. HB101 and OP50 cells are the same size (2.8±0.7 µm and 3.0±0.4 µm, respectively), but OP50 are more adhesive compared to HB101 [39].

Mutations that affect feeding generally do so by affecting the rhythmic contractions of pharyngeal muscle, resulting in decreased or arrhythmic pharyngeal pumping and therefore “inefficient” feeding. Such mutations affect either pharyngeal muscle morphology and/or function (e.g. *pha-2*, *eat-2*; [33],[40]) or the neurons that innervate the muscles (e.g. *eat-4* and *ceh-28*; [41],[42]). *hlh-6* differs from other genes involved in feeding as *hlh-6* functions in pharyngeal glands. Consistent with *hlh-6* not acting in either pharyngeal muscle or neurons, we find that *hlh-6* mutants had normal pharyngeal pumping with respect to both rate and rhythm of the muscle. Control animals (*rol-6 unc-4*) had an average of 169±39 pumps per minute (n = 20) and *hlh-6* mutants (*rol-6 hlh-6 unc-4*) had an average of 156±42 pumps per minute (n = 19). Likewise, peristaltic contractions of the pharyngeal isthmus were also normal, with both control and mutant strains showing an average of one isthmus contraction per four pharyngeal pumps. These findings indicate that *hlh-6* mutants are defective in some other aspect of food transport for which the glands are required.

### Genetic Ablation of Glands Phenocopies *hlh-6*

Because some gland genes are expressed independently of *hlh-6*, *hlh-6* mutants might be only partially impaired with respect to gland activity. To examine the effect of complete loss of pharyngeal glands, we genetically ablated the glands using an *hlh-6::egl-1* transgene, which activates expression of the pro-apoptotic gene *egl-1* in pharyngeal glands [43]. Induction of *egl-1* is sufficient to induce apoptosis in other cells, such as pharyngeal neurons [44]. We assayed the presence or absence of glands using an integrated *phat-1::YFP* reporter and followed the presence of the *hlh-6::egl-1* transgene with an intestine-specific *mTomato* marker [45]. Transgenic animals that lacked pharyngeal glands were viable but showed delayed growth and development, with 39% (n = 23) larval arrest, comparable to *hlh-6* mutants (data not shown). These results suggest that the pharyngeal glands of *C. elegans* are primarily involved in efficient feeding and that in the absence of *hlh-6*, glands are entirely nonfunctional with respect to growth and fecundity.

### Pharyngeal Glands Secrete Mucin-Related Proteins that Line the Pharyngeal Cuticle

By analogy to foregut glands in other organisms, we postulated that pharyngeal glands could function in feeding by one of three ways: first, glands may secrete digestive enzymes required for efficient feeding; second, glands may produce secretions that coat food to ensure its passage along the lumen; third, glands may produce secretions that line the lumen and prevent adhesion of food. The first possibility, that the glands produce digestive enzymes, was suggested in part by the fact that the gland-expressed gene *lys-8* is predicted to encode a lysozyme [22]. However, the ability of HB101 bacteria to rescue the starvation phenotype of *hlh-6* animals suggests that glands are not required for digestion of food.

The other two possibilities, in which the glands lubricate the pharyngeal lumen, were suggested by the ability of a less sticky food source (HB101) to rescue *hlh-6* starvation. As noted, the majority of known gland-expressed genes are predicted to encode secreted proteins that contain multiple copies of the ShK domain. Interestingly, this family of proteins is similar to a group of secreted mucins from the parasitic nematode *Toxocara canis* [46]–[48]. The *T. canis* mucins are defined by multiple copies of the ShK domain (sometimes referred to as the SXC domain), a signal sequence and stretches of Ser/Thr-rich (probable sites of glycosylation). We find that, like the *T. canis* proteins, the PHAT proteins contain stretches of Ser/Thr-rich sequence between their ShK domains (Figure S4) and many of these Ser/Thr sites are predicted to be sites for O-linked glycosylation [49]. The PHAT proteins may therefore function as mucin-like proteins.

**Figure 8:**
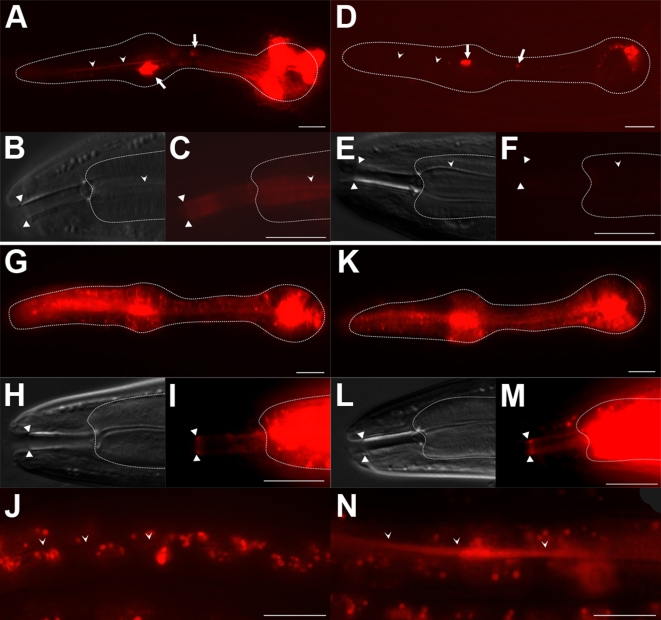
PHAT-5::MCHERRY localization in wild type and *hlh-6* mutants. Fluorescence and NDIC images of (A–C) wild-type and (D–F) *hlh-6* animals expressing the *hlh-6::phat-5::mCherry* translational fusion construct. (B) and (C) are close-ups of animal shown in (A). (E) (F) are close-ups of (D). Fluorescence and NDIC images of (G) wild-type and (K) *hlh-6* animals expressing the *myo-2::phat-5::mCherry* translational fusion construct with corresponding close-ups in (H–I) and (L–M). Arrowheads indicate the pharyngeal lumen, arrows mark the processes of the g1 glands and triangles mark the boundary of the pharyngeal cuticle. PHAT-5::MCHERRY is not found in the intestinal lumen of wild type animals (J) but is present in the intestinal lumen of *hlh-6* mutants (N), indicated by carats. Anterior is at left and the pharynx is outlined. Scale bars represent 10 µm.

We found that a representative PHAT protein, PHAT-5, lines the pharyngeal lumen, consistent with the protein having a mucin-like function. We examined the subcellular location of PHAT-5 using a *phat-5::mCherry* fusion expressed under the control of the *hlh-6* promoter. The PHAT-5::MCHERRY fusion protein was visible in discrete puncta throughout the cell bodies of the glands, as well as along their extensions (Figure 8A). In live animals, these puncta could be seen to traffic along the extensions, suggesting that the protein had been packaged into secretory vesicles. More importantly, the PHAT-5:: MCHERRY fusion protein was found along the lumen of the pharynx, indicating that the protein had been secreted from the glands (Figure 8A–C). The fusion protein had a discrete anterior boundary, extending as far as the cheilostom groove in the buccal cavity (Figure 8B,C), the boundary between the epidermal cuticle and the pharyngeal cuticle [50], suggesting that PHAT-5 is specifically associated with pharyngeal cuticle. In addition, PHAT-5 fusion protein remained associated with shed pharyngeal cuticle, arguing that the protein forms part of the lining of the pharyngeal lumen (Figure S6). No protein was seen to co-localize with bacteria in the pharynx lumen, suggesting that PHAT-5 does not coat food particles.

To investigate whether the glands of *hlh-6* mutants are functionally impaired, we examined whether PHAT-5::MCHERRY could be secreted by the glands of *hlh-6* mutants. *phat* gene expression is absent from *hlh-6* animals, so we expressed the PHAT-5 fusion under the control of the *hlh-6* promoter, which remains active in *hlh-6* mutants. The *hlh-6::phat-5::mCherry* construct was expressed in pharyngeal glands, but no protein was seen at the pharyngeal lumen, likely reflecting a functional defect in the *hlh-6* glands (Figure 8D–F). No rescue of the *hlh-6* phenotype by *hlh-6::phat-5::mCherry* was observed. Punctate signal was observed in the gland ducts and in live animals these puncta appeared to migrate along the ducts as in wild type, suggesting that vesicles were still present and capable of being transported within the glands. The *hlh-6* mutants are therefore defective either in secretion of the PHAT-5 protein or in retention of this protein at the pharyngeal lumen. To distinguish between these possibilities we expressed PHAT-5::MCHERRY in pharyngeal muscles (using the *myo-2* promoter; [51]) to investigate the localization of PHAT-5 independent of gland function. In wild type animals, pharyngeal muscle could secrete PHAT-5::MCHERRY. Signal was seen lining the pharyngeal cuticle in addition to puncta throughout the muscles (Figure 8G–I). In *hlh-6* mutants, some signal was visible on the luminal surface, but we also observed significant signal in the intestinal lumen (though not associated with cell surfaces), which was not observed in wild type animals (Figure 8J–M). This result suggests that while PHAT-5::MCHERRY can associate with the pharyngeal cuticle in *hlh-6* animals, this association is less stable, resulting in the movement of the fusion protein along the digestive tract. This observation is consistent with the hypothesis that the pharyngeal lining is defective in *hlh-6* mutants, likely due to the absence of other gland-secreted proteins, including the other PHAT proteins. No rescue of the *hlh-6* phenotype by *myo-2::phat-5::mCherry* was observed.

## Discussion

Based on our findings, we propose that HLH-6 regulates a battery of pharyngeal gland-expressed genes in *C. elegans* and is required for both differentiation and function of the glands. While some glands are present in *hlh-6* mutants, they are non-functional, as the removal of pharyngeal glands phenocopies the loss of *hlh-6*. The pharyngeal glands are essential for efficient feeding and appear to play a role in facilitating the transport of bacteria along the pharyngeal lumen, though they are not involved in regulation of pharyngeal pumping. These findings illustrate a previously unknown role for the pharyngeal glands in efficient feeding and demonstrate that aspects of both foregut gland development and function are evolutionarily conserved.

### PGM1 and HLH-6 Are Required for Gene Expression in Pharyngeal Glands

We identified both a *cis*-regulatory element and *trans*-acting factor that are required for expression in pharyngeal glands, though it is presently not known whether the two components interact directly. There are two lines of evidence that support the hypothesis that HLH-6 interacts directly with PGM1. First, the PGM1 motif contains a functional E-box (Figure 1), and bHLH proteins (like HLH-6) bind to E-boxes. Second, PGM1 activity requires HLH-6 (Figure 4). A formal possibility is that HLH-6 acts upon a second bHLH that in turn binds to PGM1, as seen with the cascades of neurogenic and myogenic bHLH factors [52],[53]. However, no other *C. elegans* class II bHLH is known to be expressed in pharyngeal glands, though some *hlh* genes remain uncharacterized.

As with other bHLH proteins, HLH-6 probably functions as a dimer, most likely with the broadly-expressed Class I protein HLH-2 [26]. However, HLH-6 appears to require an additional non-bHLH factor that functions through the YMAAY sequence found in PGM1. Three lines of evidence indicate that HLH-6 requires additional factor(s) to activate gland gene expression. First, the YMAAY sequence is required for PGM1 activity, but is unlikely to represent an extended binding sequence for HLH-6, as solved bHLH-DNA structures indicate contact of bHLH proteins up to but not beyond three bases outside of the E-box [54],[55], while the YMAAY sequence extends beyond this limit. Second, ectopic expression of HLH-6 (±HLH-2) is not sufficient to activate ectopic expression of a gland-expressed marker (data not shown), suggesting that an additional factor is required to induce target gene expression. Third, we tested whether HLH-6 (±HLH-2) could bind to PGM1 *in vitro* using electrophoretic mobility shift assays (EMSA), but were unable to detect an interaction (Text S1 and Figure S5), though we are able to detect interactions between other bHLH dimers and E-box-containing sequences. Thus, the YMAAY sequence likely represents a binding site for an additional factor. This factor may be limiting with respect to activation of gland genes *in vivo* and binding to PGM1 *in vitro*. Precedence for such a model comes from studies of mammalian Mash1, which must form a complex with the POU domain transcription factor Brn2 in order to bind to specific target sequences [56]. Similarly, the pancreatic determinant PTF1 is a complex of the bHLH Ptf1a with a ubiquitous Class I bHLH and the mammalian Su(H) ortholog RBP-J [57]; the PTF1 complex binds to a composite DNA sequence consisting of an E-box and a Su(H) site [58].

Involvement of an additional factor may explain the specificity of PGM1 activity. A general question in transcription factor biology is how specificity of response is achieved. For example, the E-box of PGM1 could be recognized by any of the numerous bHLH factors expressed in the various tissues of *C. elegans*, yet it is only activated in pharyngeal glands (Figure 2). One solution to this problem is that related transcription factors distinguish between different binding sites based on subtle differences within the core DNA sequence. For example, different MyoD-containing bHLH dimers have well-characterized binding site preferences [59],[60], as do the *C. elegans* bHLH factor Twist/HLH-8 [28],[61] and the *Drosophila* bHLHs *atonal* and *scute* [62]. However, given that binding of bHLH factors to E-boxes may be somewhat promiscuous *in vitro*, an additional approach to ensure specific response is the involvement of spatially restricted co-factors. Tertiary interactions between bHLH dimers and non-bHLH co-factors are known to affect dimerization and activity [63],[64]. In our case, a cofactor may recognize the YMAAY portion of PGM1 and be required for transcriptional activation of target genes.

### HLH-6 and the Pharyngeal Gene Network

The FoxA transcription factor PHA-4 is required for specification of pharyngeal cells, including glands [1]. One question, then, is the regulatory relationship between PHA-4 and the HLH-6 gene battery. We have shown in other work that HLH-6 is a probable direct target of PHA-4, so PGM1-dependent genes are at least indirectly regulated by PHA-4 [30]. However, previous work suggested that most or all pharyngeal genes are directly regulated by PHA-4 [4]. Consistent with this idea, we find candidate PHA-4 binding sites in the regulatory regions of all seven gland genes analyzed in addition to the PGM1 motif (Figure S1). Furthermore, a deletion of the *phat-1* promoter that removes a predicted PHA-4 binding site drastically reduces but does not eliminate reporter expression and does not affect the pattern of expression (data not shown). Similar results are seen with the PHA-4 sites in other promoters (e.g. *myo-2*; [4]). PHA-4 may regulate gland-specific gene expression both directly and indirectly, consistent with the proposed model of PHA-4 action. This type of feed-forward transcriptional regulation is also observed in other developmental pathways, such as the myogenic cascade of bHLH transcription factors [53].

### Other Factors Required for Gland Development

*hlh-6* mutants have multiple defects in gland differentiation, yet still produce g1 (and occasionally g2) gland-like cells and express at least some gland-specific markers (such as *B0507.1* and *Y8A9A.2*; Figure 4, Figure S2). Therefore, different factors activate expression of different gene batteries in pharyngeal glands, as occurs in body wall muscles and in the excretory cell of *C. elegans* [65],[66]. It will be interesting to identify more HLH-6-independent genes to determine whether the function of that gene battery is distinct from the role of the HLH-6-dependent gene battery; that is, are the different functions of the cells parsed out in an interpretable manner?

bPrevious work suggested a role for pharyngeal glands in feeding, based on analysis of the *kel-1* gene [11]. KEL-1 is detected in pharyngeal glands and *kel-1* mutants arrest as early larvae and fail to reach adulthood, in contrast to *hlh-6* mutants and gland-ablated animals which are starved but viable. One possible explanation for the difference in phenotypes is that *kel-1* function is not limited to pharyngeal glands. In fact, available *in situ* hybridization data for *kel-1* indicates that the message is broadly expressed throughout embryogenesis, with no apparent enrichment in glands [15],[16]. Thus, loss of *kel-1* likely affects cells in addition to the pharyngeal glands.

### Specification and Differentiation of Pharyngeal Glands

The defects in *hlh-6* mutants are consistent with HLH-6 playing a role in differentiation of gland cells rather than their specification. The g1 cells still have several “gland-like” features in *hlh-6* mutants: the cell bodies are located in the terminal bulb, they express some gland-specific markers and the cells send projections to the appropriate positions within the pharynx. However, these cells are not fully functional as ablation of all the gland cells is no more severe than loss of *hlh-6* alone, indicating that the residual glands in *hlh-6* mutants contribute little, if any, wild type function. The g2 gland defect is more pronounced as these cells fail to differentiate in *hlh-6* mutants, apparently arresting as precursor cells with an uncertain identity. These cells lose expression of *pha-4::GFP::HIS2B*, suggesting that they fail to retain pharyngeal identity. A similar loss of *pha-4* reporter expression in seen in *tbx-2* mutants, which fail to produce anterior pharyngeal muscle [8]. An interesting possibility is that successful differentiation of pharyngeal cells (into specific cell types) is required for maintenance of *pha-4* expression and pharyngeal identity. A similar loss of cell identity may occur in *unc-120*; *hlh-1*; *hnd-1* triple mutants, in which presumptive body muscles are found in their normal position within the embryo yet do not adopt a muscle identity nor do they adopt an alternate (non-muscle) fate [66]. In contrast, *C. elegans* neurons that lose specific sub-type identities retain their neuronal identity [67].

Although we do not detect *hlh-6* expression in the g2 precursors of wild-type animals, it must be expressed at this time, as *hlh-6* activity is required for division of the precursors and g2 development can be rescued by transgenic *hlh-6(+)*. The relevant expression is likely to be too weak to be detectable.

### The HLH-6 Gene Battery and Pharyngeal Gland Function

The pharyngeal glands are required for efficient feeding (Figures 6 and 7). A compelling model is that the glands secrete material that coats the pharyngeal lumen to prevent food from adhering to the pharyngeal cuticle. Support for this model comes from three lines of evidence. First, *hlh-6* mutants are feeding defective yet have normal pharyngeal pumping. Second, the starvation phenotype of *hlh-6* mutants is rescued by feeding with a different (less sticky) food source. Third, the lining of the pharynx differs in *hlh-6* mutants as shown by the inability of the secreted PHAT-5::MCHERRY protein to adhere tightly to the pharyngeal lumen as it does in wild type (Figure 8). Many of the HLH-6-dependent gland genes encode mucin-like proteins, at least one of which (PHAT-5) lines the pharyngeal cuticle. Although we did not demonstrate that PHAT's are responsible for lubrication, we propose a speculative model in which gland secretion of the mucin-related PHAT proteins act to lubricate the pharyngeal lining, comparable to some aspects of mucin function in other organisms [17].

The positioning of the gland duct openings at discrete points along the length of the pharyngeal lumen could also be explained by a requirement for thorough lining of the lumen, as mosaic animals that express a PHAT-5 fusion in only a subset of glands show incomplete coverage of the pharyngeal lining. For example, when a PHAT-5::YFP fusion is expressed in only the g1P cell, which opens at the anterior end of the pharynx, fluorescent signal is detectable at high levels at the anterior end of the pharynx but decreases posteriorly, becoming undetectable before the terminal bulb (Figure S6). Likewise, expression in only the g1A cells results in signal near the middle of the pharynx that fades towards the anterior and posterior extremes. Thus, secretion from all five glands may be required for complete lining of the pharyngeal lumen.

### Evolutionary Conservation of Foregut Gland HLH Genes

An interesting finding is that both the regulation (by bHLH factors) and function (feeding) of foregut glands appears to be evolutionarily conserved. The closest mammalian homolog of HLH-6 is Sgn1, a bHLH required for normal salivary gland development in the mammalian foregut [68]. In addition, development of salivary glands in the *Drosophila* foregut depends on the combined activity of *forkhead* (the ortholog of PHA-4) and *sage* (a salivary gland expressed bHLH) [69], although *sage* is not the closest homolog to *hlh-6*. Database searches have found other genes encoding proteins with high similarity to HLH-6, including the *Ash2* gene, which is expressed in the digestive tract glands of the jellyfish *P. carnea* [70], and related sequences from the genomes of parasitic nematodes. Gland function in parasitic nematodes is critical for parasitism, suggesting a conserved function of foregut glands in the processing or passage of food [46],[71]. Targeting gland development or function may offer a new strategy for controlling these parasitic species.

## Materials and Methods

### Worm Strains

Standard nematode handling conditions were used [72]. The *hlh-6(tm299) II* allele was kindly provided by S. Mitani [29]. Presence of the *tm299* deletion was followed by genomic PCR with oligonucleotides oGD65 (5′ CATAACCGGTATCATAGCATTATTACTCGAAT 3′) and oGD97 (5′ TTATACATTTGAGAATGGGGTCTACTCGAC 3′). The original *hlh-6(tm299)*-bearing chromosome contains a linked larval lethal mutation (*let-x*) to the left of *hlh-6*. *hlh-6* was outcrossed five times and the arms of LG II were replaced by selecting appropriate recombinants tested for the presence of *hlh-6(tm299)* by PCR. First, we placed *unc-4* in *cis* with *let-x hlh-6* and then selected Rol non-Daf recombinants from *let hlh-6 unc-4/rol-6(e187) daf-19(m86)* to obtain *+rol-6 hlh-6 unc-4*. Because this strain is Rol Unc, in all subsequent functional assays a *rol-6 unc-4* strain was used as a control.

### Construction of Plasmids

All transcriptional reporters were made by PCR amplification of promoter fragments from genomic DNA, followed by cloning into either the pPD95.77 or pPD95.77-YFP vectors (gifts from A. Fire), which contain the coding sequences for *gfp* and *yfp*, respectively. Mutations in occurrences of PGM1 in the promoters were subsequently made by PCR-based site-directed mutagenesis [73].

Enhancer constructs were built using synthetic oligonucleotides that were cloned into pPD95.77. Use of this vector for enhancer assays was established previously [67].

The 750 base pair *phat-5* cDNA was amplified from a cDNA library provided by R. Barstead using primers oGD570 (5′ aaggtacccATGGTGAGCAAGGGCGAG 3′) and oGD571 (5′ ccgaattcTTACTTGTACAGCTCGTCCATGCC 3′). The product was digested with enzymes *Kpn*I and *Eco*RI (restriction sites in the oligonucleotides are underlined), and cloned in-frame to *YFP* or *mCherry* [45]. The *phat-5::YFP* fusion was placed under the control of the *lys-8* promoter, while the *hlh-6* minimal promoter was sub-cloned from *min-hlh-6::YFP* [30] in front of the *phat-5::mCherry* fusion to create the *hlh-6::phat-5::mCherry* construct. The *myo-2::phat-5::mCherry* plasmid was cloned using the *myo-2* promoter from plasmid pSEM474 [4].All clones were verified by restriction digests and sequencing. Details of plasmids and cloning strategies are available upon request.

For rescue of *hlh-6* mutants, we subcloned a 3398 bp *Pst*I-*Xba*I fragment of fosmid WRM066cG05 that contains *hlh-6(+)* into pBlueScriptII(SK+). The “minigene” construct was created by amplification and subcloning of the *hlh-6* cDNA from a library provided by R. Barstead. The cDNA was ligated to a 568 bp fragment of the *hlh-6* promoter that is active in pharyngeal glands [30]. A synthetic intron was cloned in to a blunt-ended *Kpn*I site of the *hlh-6* cDNA using annealed primers oGD198 (5′ GgtaagtttaaacagatatctactaactaaccctgattatttaaattttcagTAC 3′; intron sequence in lower case) and oGD199 (5′ GTActgaaaatttaaataatcagggttagttagtagatatctgtttaaacttacC 3′).

The *hlh-6::egl-1* plasmid was constructing by PCR amplification of *egl-1* from genomic N2 DNA using primers oGD531 (5′ caccaccggtatgctggtaagtctagaaattatt 3′) and oGD532 (5′ ttcacggccgcacatctggtgttgcaggc 3′). The amplified product was digested with *Age*I and *Eag*I and cloned downstream of a 747 bp fragment of the *hlh-6* promoter. Design of this construct was based on previous work [44].

### Construction of Transgenic Lines

Reporter DNA was injected at 5–30 ng/µL together with 50 ng/µL pRF4 (*rol-6(su1006)*), which confers a dominant Roller phenotype [74], and 20–45 ng/µL pBS II (SK+) to a total DNA concentration of 100 ng/µL. For some analyses, we included 20 ng/µL of an intestine specific reporter (*elt-2::GFP::LacZ*, *ges-1::mRFP::His2B* or *elt-2::mTomato::HIS2B*) that served as an independent marker for transgenic arrays when scoring expression [75]. For injections with enhancer constructs, 50 ng/µL of the construct was injected with 50 ng/µL pRF4 into N2 animals. For *hlh-6::phat-5::mCherry*, 40 ng/µL was injected while *myo-2::phat-5::mCherry* was injected at 5 ng/µL, because the *myo-2* promoter is very strong and can be toxic at higher concentrations. Except where noted, a minimum of two independent transgenic lines were analyzed for each construct.

The integrated *hlh-6* reporter *ivIs10* [*hlh-6*::*YFP ges-1*::*mRFP::His2B rol-6(su1006*)] and integrated *phat-1::YFP* reporter *ivIs12* [*phat-1::YFP elt-2::GFP::LacZ rol-6(su10060*] were generated by gamma-ray-induced integration of extrachromosomal arrays carried in a wild-type background [76]. The *pha-4::GFP::HIS2B* reporter was provided by Dr. Susan Mango as an integrated array (SM496), which was crossed into the GD211 strain.

To induce cell death in glands, the *hlh-6::egl-1* construct was injected at 20 ng/µL with 30 ng/µL *elt-2::mTomato::HIS2B* and 50 ng/µL pBS II (SK+) into a strain carrying an integrated *phat-1::YFP* reporter (GD139 *ivIs12*; see above). Doubly transgenic animals were identified based on the Rol phenotype of GD139 (100%) and the presence of red intestinal fluorescence. Animals lacking visible YFP expression (indicating a loss of glands) were then analyzed for survival and growth.

For rescue of *hlh-6*, both the genomic fragment and the minigene were injected at 50 ng/µL with 30 ng/µL of *phat-1::YFP* and 20 ng/µL *elt-2::GFP::LacZ* into N2 animals. These arrays were subsequently crossed into GD211.

### Motif Searches using Improbizer

We used the Improbizer program [14]; available at http://www.soe.ucsc.edu/˜kent/improbizer/) to search for possible gland-specific regulatory elements. We initially searched for motifs occurring once per sequence, using the input sequence as background. The motif presented here (PGM1) was obtained with a search for a motif size of six. Searches for motifs of larger sizes (8–20 bases) recurrently found variations of PGM1. Other parameters of Improbizer were used at their default settings. We also performed control runs in which the input gene sequence was randomized and searched and found that only PGM1 obtained an Improbizer score greater than the scores of ten or more control runs.

To find probable occurrences of PGM1 in other promoters (as in *pqn-8* and *lys-8*), we used the Improbizer sister program, Motif Matcher (www. http://www.cse.ucsc.edu/˜kent/improbizer/motifMatcher.html), which searches for top-scoring matches to the Improbizer-generated position weight matrix.

### Cell Lineage Analysis

Lineages of embryos from *hlh-6/mC6g* heterozygotes or *hlh-6* homozygotes were examined using a 4D-microscope [77]. The genotype of *hlh-6/mC6g* progeny was determined after recording by the presence or absence of GFP, which marks the *mC6g* balancer chromosome. The identities of cells was determined by lineaging backwards using the data base SIMI°Biocell.

### Growth Assays

All animals were grown on OP50 except for the OP50-GFP bacteria used to visualize the stuffed pharynx and the HB101 bacteria used to rescue the *hlh-6* mutant; all bacterial strains were provided by the *Caenorhabditis* Genetics Center. OP50-GFP was grown on NGM plates containing 100 µg/mL ampicillin and HB101 was grown on NGM plates containing 200 µg/mL streptomycin.

For measurement of body length, embryos laid over a one hour period by gravid adults were collected from and grown at 25°. Larvae were removed from plates and transferred to slides at the indicated times. Pictures were taken at 400× magnification and the lengths of the animals were measured using ImageJ (http://rsb.info.nih.gov/ij/) as described previously [34]. Greater than twenty animals were analyzed for each genotype at each time-point. For measuring time to reach adulthood, single eggs were placed on plates and followed at 24 hour intervals until the animal reached adulthood. For brood sizes the number of eggs laid was counted throughout the lifetime of each animal.

The intestinal fat stores of the *hlh-6* mutants were measured using the dye Nile Red (Sigma N-3013) as described [35]. Briefly, L4 animals of the indicated genotype were transferred to plates with 0.05 µg/mL Nile Red and allowed to grow for 24 hours before being scored using conventional fluorescence microscopy. At least fifteen animals were observed for each genotype and one animal that represents the average level of fluorescence per each genotype is shown. *daf-16(RNAi)* was performed by “feeding RNAi” using an available *daf-16* dsRNA-expressing bacterial strain [78],[79]. Adults were placed on the RNAi plates and their progeny were transferred to RNAi-Nile Red plates for scoring.

For pharyngeal pumping assays, L4 animals were transferred to fresh plates and grown for 24 hours before scoring. Pumping was counted under a dissecting microscope at 100× magnification.

## Supporting Information

Figure S1 Scale diagrams of the analyzed gland-specific promoters. Triangles indicate candidate PHA-4 binding sites (TRTTKRY) and black rectangles indicate occurrences of PGM1. The grey rectangle represents a weak occurrence of a functional PGM1.
(0.56 MB TIF)

Figure S2 Representative images of the (A) *phat-3*, (B) *phat-5*, (C) *B0507.1*, (D) *pqn-8* and (E) *lys-8* reporters in *hlh-6* mutant animals. Expression of *Y8A9A.2::GFP* in (F) wild type and (G) *hlh-6* mutants. Anterior is at left and the pharynx is outlined. Scale bars represent 10 µm.
(1.10 MB TIF)

Figure S3 Pharyngeal cell type-specific markers were examined to determine if the g2 cells had adopted an alternate pharyngeal cell fate. We used the pan-neuronal *rgef-1::GFP* marker to count pharyngeal neurons (expect 7 in wild type), *myo-2::GFP::His2B* to count pharyngeal muscle nuclei (expect 4 in wild type) and *pax-1::GFP::His2B* to count pharyngeal marginal cell nuclei (expect 4 in wild type, as *pax-1::GFP* is also expressed in the pm8 muscle) [51],[81],[82]. We saw no change in the number of cells expressing these three markers in wild-type animals and *hlh-6* mutants (6.6 vs 7.0 neurons, 3.6 vs. 3.7 muscles and 3.7 vs. 3.6 marginal cells, respectively). Error bars are standard deviation.
(1.06 MB TIF)

Figure S4 (A) *C. elegans* PHAT-1 and *T. canis* MUC-5 [47] protein sequences showing predicted signal sequence (highlighted in yellow), ShK motifs (red) and Ala/Ser/Thr-rich tracts predicted to contain O-glycosylation sites (underlined). Signal sequences predicted using SignalP 3.0 [83]. (B) PHAT-1 contains numerous predicted O-glycosylation sites that lie between the ShK motifs. Generated using the NetOGlyc 3.1 server [49].
(2.86 MB TIF)

Figure S5 Electrophoretic mobility shift assay of PGM1. Lane 1 is free probe, Lane 2 is probe with unprogrammed reticulocyte lysate. Lanes 6 and 8 are reactions in which the two proteins were independently transcribed and translated. Lanes 7 and 9 are reactions in which the two proteins were co-translated. Other lanes are as indicated. Open arrow indicates free probe, thin arrow indicates non-specific shift obtained with reticulocyte lysate alone, black arrow indicates HLH-2+HLH-3 shift. No HLH-2+HLH-6 shift is observed, though expression of both proteins has been verified by ^35^S-Met labeling (not shown).
(0.53 MB TIF)

Figure S6 Expression of *phat-5::mCherry* constructs in wildtype animals. (A) Expression of *hlh-6::phat-5::mCherry* that is weaker than that shown in Figure 8a for a comparison with the decreased levels in the *hlh-6* mutant. The pharyngeal lumen is indicated by arrowheads. (B,C) Expression of *lys-8::phat-5::YFP* with a random loss of the reporter in subsets of glands. (B) Loss of the reporter in g1P, g2L and g2R so that only g1AL and g1AR express the fusion construct. (B) Loss of the reporter in g1AL, g1AR, g2L and g2R so that only g1P expresses the construct. Arrows indicate the boundary of PHAT-5::YFP attachment to the pharyngeal lumen. (D,E) Expression of *lys-8::phat-5::YFP* during the L1 to L2 molt. The expelled buccal cavity cuticle is indicated by an asterisk and the boundary of the new buccal cuticle is indicated by triangles. Anterior is at left and the pharynx is outlined. Scale bars represent 10 µm.
(1.08 MB TIF)

Table S1 Lists of gland and pharyngeal (non-gland) genes and their associated Motif Matcher score. The gland list is as in Table 1, the non-gland list is a list of previously identified microarray positives with supporting expression data [27]. Motif Matcher scores were generated using the computationally identified PGM1 run against 500 bp of sequence upstream sequence (relative to the ATG) for each of the indicated genes. Motif Matcher is the sister program to Improbizer and is available at http://www.soe.ucsc.edu/˜kent/improbizer/motifMatcher.html [14]. Scores over 7.00 were considered to be good matches to PGM1, consistent with our functional characterization of the motif. Given that this threshold score is somewhat arbitrary, we also examined the difference between the scores for the two gene sets using the Mann-Whitney U test and found that gland genes had a significantly higher PGM1 score than did non-gland genes (P<0.001).
(0.04 MB DOC)

Text S1 Supplemental materials.
(0.02 MB DOC)
