# Supplementary figures and images for: The HLH-6 Transcription Factor Regulates C. elegans Pharyngeal Gland Development and Function
Source: PLoS Genet. 2008 Oct 17;4(10):e1000222. doi: 10.1371/journal.pgen.1000222 (PMC2563036; doi:10.1371/journal.pgen.1000222)

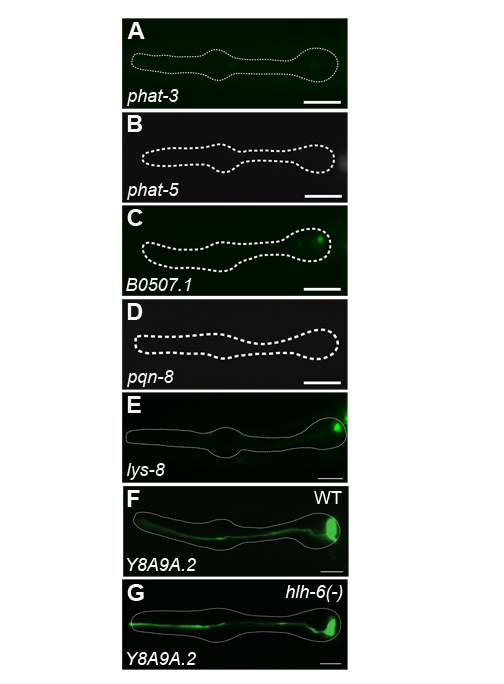

Supplement: Figure S2 — Representative images of the (A) phat-3, (B) phat-5, (C) B0507.1, (D) pqn-8 and (E) lys-8 reporters in hlh-6 mutant animals. Expression of Y8A9A.2::GFP in (F) wild type and (G) hlh-6 mutants. Anterior is at left and the pharynx is outlined. Scale bars represent 10 µm. (1.10 MB TIF) [file pgen.1000222.s002.tif]

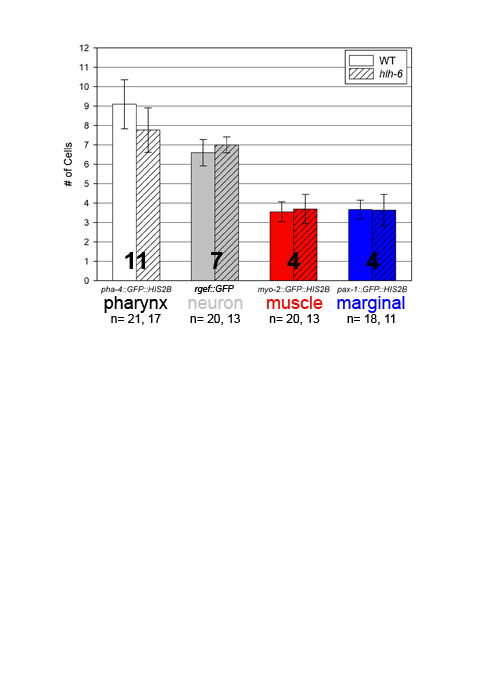

Supplement: Figure S3 — Pharyngeal cell type-specific markers were examined to determine if the g2 cells had adopted an alternate pharyngeal cell fate. We used the pan-neuronal rgef-1::GFP marker to count pharyngeal neurons (expect 7 in wild type), myo-2::GFP::His2B to count pharyngeal muscle nuclei (expect 4 in wild type) and pax-1::GFP::His2B to count pharyngeal marginal cell nuclei (expect 4 in wild type, as pax-1::GFP is also expressed in the pm8 muscle) [51],[81],[82]. We saw no change in the number of cells expressing these three markers in wild-type animals and hlh-6 mutants (6.6 vs 7.0 neurons, 3.6 vs. 3.7 muscles and 3.7 vs. 3.6 marginal cells, respectively). Error bars are standard deviation. (1.06 MB TIF) [file pgen.1000222.s003.tif]

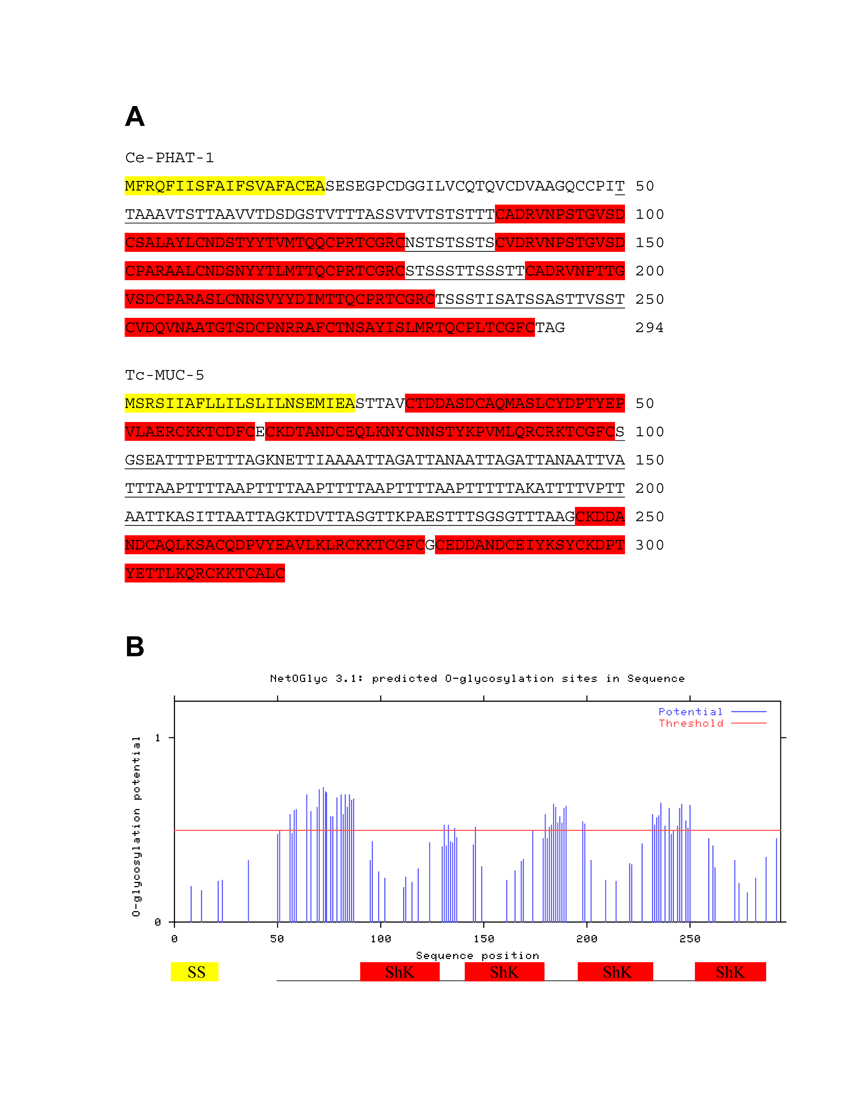

Supplement: Figure S4 — (A) C. elegans PHAT-1 and T. canis MUC-5 [47] protein sequences showing predicted signal sequence (highlighted in yellow), ShK motifs (red) and Ala/Ser/Thr-rich tracts predicted to contain O-glycosylation sites (underlined). Signal sequences predicted using SignalP 3.0 [83]. (B) PHAT-1 contains numerous predicted O-glycosylation sites that lie between the ShK motifs. Generated using the NetOGlyc 3.1 server [49]. (2.86 MB TIF) [file pgen.1000222.s004.tif]

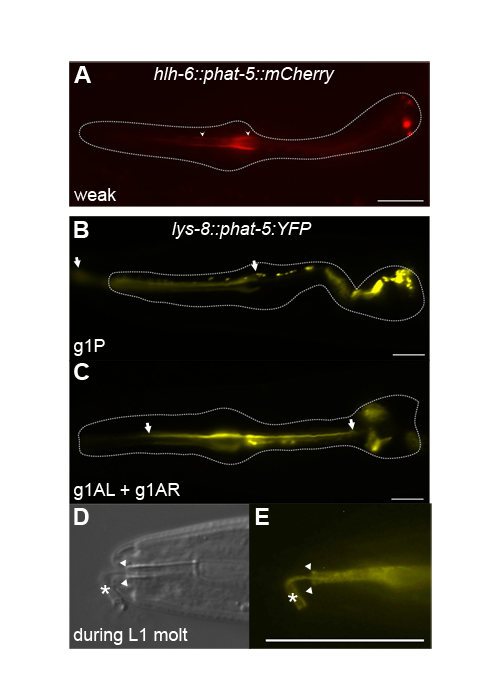

Supplement: Figure S6 — Expression of phat-5::mCherry constructs in wildtype animals. (A) Expression of hlh-6::phat-5::mCherry that is weaker than that shown in Figure 8a for a comparison with the decreased levels in the hlh-6 mutant. The pharyngeal lumen is indicated by arrowheads. (B,C) Expression of lys-8::phat-5::YFP with a random loss of the reporter in subsets of glands. (B) Loss of the reporter in g1P, g2L and g2R so that only g1AL and g1AR express the fusion construct. (B) Loss of the reporter in g1AL, g1AR, g2L and g2R so that only g1P expresses the construct. Arrows indicate the boundary of PHAT-5::YFP attachment to the pharyngeal lumen. (D,E) Expression of lys-8::phat-5::YFP during the L1 to L2 molt. The expelled buccal cavity cuticle is indicated by an asterisk and the boundary of the new buccal cuticle is indicated by triangles. Anterior is at left and the pharynx is outlined. Scale bars represent 10 µm. (1.08 MB TIF) [file pgen.1000222.s006.tif]
