# Supplementary material for: The HLH-6 Transcription Factor Regulates C. elegans Pharyngeal Gland Development and Function
Source: PLoS Genet. 2008 Oct 17;4(10):e1000222. doi: 10.1371/journal.pgen.1000222 (PMC2563036; doi:10.1371/journal.pgen.1000222)
Supplement: Table S1 — Lists of gland and pharyngeal (non-gland) genes and their associated Motif Matcher score. The gland list is as in Table 1, the non-gland list is a list of previously identified microarray positives with supporting expression data [27]. Motif Matcher scores were generated using the computationally identified PGM1 run against 500 bp of sequence upstream sequence (relative to the ATG) for each of the indicated genes. Motif Matcher is the sister program to Improbizer and is available at http://www.soe.ucsc.edu/˜kent/improbizer/motifMatcher.html [14]. Scores over 7.00 were considered to be good matches to PGM1, consistent with our functional characterization of the motif. Given that this threshold score is somewhat arbitrary, we also examined the difference between the scores for the two gene sets using the Mann-Whitney U test and found that gland genes had a significantly higher PGM1 score than did non-gland genes (P<0.001). (0.04 MB DOC) [file pgen.1000222.s007.doc]

**Supplemental Table 1**

Gland genes

10.85 T05B4.11

10.85 C49G7.4

10.83 T20G5.7

10.83 F07C4.11

10.83 B0507.1

9.27 C46H11.9

8.45 T05B4.3

8.43 T05B4.8

8.43 C46H11.8

7.96 F41G3.10

7.57 M153.3

7.50 T05B4.13

6.30 T10B10.6

5.09 T05B4.12

Pharyngeal, non-gland genes

10.32 T06E4.11

9.84 F35A5.3

9.16 C10G8.5a

8.97 F08B12.1

8.90 K08F8.2

8.47 T05E11.3.1

8.45 M03D4.4a

8.42 T22B2.6

8.29 C01B10.5a

8.24 Y76A2B.2

8.02 DY3.5

7.93 T06E4.9

7.81 F49E10.2a

7.80 R07B1.9

7.64 T23F6.1

7.45 T10E10.4

7.35 C23H3.9a

7.26 C03A7.14

7.09 F13H8.4

7.07 F25G6.6

7.00 F54F3.1

6.89 R03C1.1

6.89 C14C11.8

6.84 C06G1.2

6.78 T27C5.10

6.78 M01D1.2a

6.78 K06A1.3

6.78 F16B4.8

6.73 F19G12.7

6.68 T04H1.6

6.61 ZK892.7

6.51 F10G8.8

6.42 C03A7.7

6.34 T04C9.4a

6.31 R09B5.5

6.28 T21C9.9

6.11 C14B9.2.1

5.98 F22A3.1

5.98 F21D5.9

5.96 F07H5.8

5.93 ZK816.4

5.85 T06E4.7

5.83 F12F3.1a.1

5.76 T06E4.8

5.75 F21H11.3.1

5.66 CD4.9

5.64 M02G9.1

5.59 R09E10.5

5.53 F54E2.3a

5.39 F58G4.1

5.29 T04C9.6a.1

5.27 F53H4.5

5.25 F11E6.8

5.23 ZK418.3

5.10 C03A7.8

4.99 F48E3.8a

4.98 F40E10.5

4.97 C04E6.12

4.94 F26A10.2

4.68 D1054.9a

4.67 T01D1.6

4.58 C03A7.4

4.53 R07E3.2

4.51 F54E2.2

4.48 T23F4.4

4.37 T23F1.6

4.30 F53A9.3

4.02 R09F10.7

4.02 R09F10.2

4.00 ZC250.1

3.97 F57B1.6

3.92 R02F11.1

3.91 T06E4.10

3.88 C44H4.1

3.82 T06D8.3

3.81 E01G6.1

3.72 D1009.5

3.68 ZK1025.7

3.66 D2024.4

3.62 T18D3.4

3.24 F45G2.2a

3.20 F14B4.1

3.04 K02F6.4

3.02 F53B3.3

2.93 F09F7.8

2.86 K11H12.5

2.78 R07C3.5

2.78 F38B6.1

2.65 F35C5.10

2.53 W10D9.1

2.49 C32H11.5

2.34 W01C9.1

2.15 M195.2

2.07 F20B10.3

1.44 ZK1067.7

1.19 C27A2.5
