# Supplementary material for: The HLH-6 Transcription Factor Regulates C. elegans Pharyngeal Gland Development and Function
Source: PLoS Genet. 2008 Oct 17;4(10):e1000222. doi: 10.1371/journal.pgen.1000222 (PMC2563036; doi:10.1371/journal.pgen.1000222)
Supplement: Text S1 — Supplemental materials. (0.02 MB DOC) [file pgen.1000222.s008.doc]

**SUPPLEMENTAL MATERIALS**

**Electrophoretic mobility shift assay**

Probe DNA oligonucleotides were annealed then end labeled with the Klenow fragment enzyme. Oligonucleotides used were oGD137 (5’ tgcaaacttgatgcacctgttcaaatgtatgccc 3’) and oGD138 (5’ tggggcatacatttgaacaggtgcatcaagtttg 3’), taken from the PGM1 site (underlined) in the promoter of *phat-3*. The Promega TnT © T7 Quick Coupled Transcription/Translation System was used to translate HLH-2, HLH-3 and HLH-6. The HLH-3 plasmid pKM1023 (His-tagged HLH-3, a gift from Dr. Michael Krause) was linearized using *EcoRI* before being transcribed/translated. PCR amplified HLH-2 and HLH-6 were obtained using the primers oGD178 (5’ gtaatacgactcactatagggcgATGgcggatccaaata 3’) and oGD179 (5’ ttaaaaccgtggatgtccaaactgc 3’) on pKM1023 (HLH-2 cDNA, a gift from Dr. Michael Krause) for HLH-2 and primers oGD244 (5’ gtaatacgactcactatagggcgccatggcaATGtcaatttcc 3’) and oGD106 (5’ gggatatctcacatagcattattactcgaat 3’) on pGD110 (heat-shock inducible HLH-6 cDNA) for HLH-6. A Kozac consensus ATG start site (underlined) was introduced before the true ATG of the HLH-6 cDNA. Binding conditions used were previously described [4] except 37 ng/μg poly (dI-dC) was used. Samples were run on 5% (w/v) native polyacrylamide gels at 100V for one hour at room temperature in a solution of 45 mM Tris, 45 mM Boric acid and 1 mM EDTA pH 8.

The pGD110 plasmid was constructed as follows. The 807 base pair *hlh-6* cDNA was amplified from a cDNA Library provided by R. Barstead using primers oGD102 (5' CGGGATCCATGTCAATTTCCCAAAACAACTT 3') and oGD103 (5' GGGGTACCTCACATAGCATTATTACTCGAAT 3'). The product was digested with enzymes BamHI and KpnI (restriction sites in the oligonucleotides are underlined), and cloned in to the heat-shock vector pPD49.83 (a gift from A. Fire).
